# Non-Invasive Modalities in the Assessment of Vulnerable Coronary Atherosclerotic Plaques

**DOI:** 10.3390/tomography8040147

**Published:** 2022-07-06

**Authors:** Panagiotis Theofilis, Marios Sagris, Alexios S. Antonopoulos, Evangelos Oikonomou, Konstantinos Tsioufis, Dimitris Tousoulis

**Affiliations:** 1 1st Cardiology Department, “Hippokration” General Hospital, Medical School, University of Athens, 11527 Athens, Greece; masagris1919@gmail.com (M.S.); antonopoulosal@yahoo.gr (A.S.A.); boikono@gmail.com (E.O.); kptsioufis@gmail.com (K.T.); drtousoulis@hotmail.com (D.T.); 2 3rd Cardiology Department, Thoracic Diseases Hospital “Sotiria”, University of Athens Medical School, 11527 Athens, Greece

**Keywords:** vulnerable plaque, atherosclerosis, coronary CT angiography, non-invasive imaging, biomarker

## Abstract

Coronary atherosclerosis is a complex, multistep process that may lead to critical complications upon progression, revolving around plaque disruption through either rupture or erosion. Several high-risk features are associated with plaque vulnerability and may add incremental prognostic information. Although invasive imaging modalities such as optical coherence tomography or intravascular ultrasound are considered to be the gold standard in the assessment of vulnerable coronary atherosclerotic plaques (VCAPs), contemporary evidence suggests a potential role for non-invasive methods in this context. Biomarkers associated with deleterious pathophysiologic pathways, including inflammation and extracellular matrix degradation, have been correlated with VCAP characteristics and adverse prognosis. However, coronary computed tomography (CT) angiography has been the most extensively investigated technique, significantly correlating with invasive method-derived VCAP features. The estimation of perivascular fat attenuation as well as radiomic-based approaches represent additional concepts that may add incremental information. Cardiac magnetic resonance imaging (MRI) has also been evaluated in clinical studies, with promising results through the various image sequences that have been tested. As far as nuclear cardiology is concerned, the implementation of positron emission tomography in the VCAP assessment currently faces several limitations with the myocardial uptake of the radiotracer in cases of fluorodeoxyglucose use, as well as with motion correction. Moreover, the search for the ideal radiotracer and the most adequate combination (CT or MRI) is still ongoing. With a look to the future, the possible combination of imaging and circulating inflammatory and extracellular matrix degradation biomarkers in diagnostic and prognostic algorithms may represent the essential next step for the assessment of high-risk individuals.

## 1. Introduction

As a leading cause of morbidity and mortality, atherosclerotic cardiovascular diseases are constantly at the forefront of scientific research to further unveil their pathophysiologic basis and to develop appropriate diagnostic and therapeutic approaches. Undoubtedly, the development of a plaque is the critical complication of atherosclerosis and the investigation of its features may guide a tailored management of patients with atherosclerotic cardiovascular diseases. In the context of coronary artery disease (CAD), significant progress has been made towards the assessment of coronary atherosclerotic plaques, since several characteristics have been identified as high-risk for rupture or erosion, leading to the development of acute coronary syndromes (ACS). According to these properties, the plaques which are prone to rupture or erosion may be defined as vulnerable plaques. Although invasive imaging methods remain the gold-standard in the evaluation of vulnerable plaques, non-invasive modalities may be critical in the early detection of those abnormalities. In this review article, we summarize the latest advances in the non-invasive assessment of vulnerable coronary atherosclerotic plaques (VCAP).

## 2. Features of the Vulnerable Coronary Atherosclerotic Plaque

The process of atherosclerosis is complex, consisting of multiple steps. The invasion of low-density lipoprotein (LDL) molecules in the subendothelial space through the aid of extracellular matrix proteoglycans and the ensuing LDL oxidation consist of the pivotal initial step [1]. Endothelial dysfunction and permeability are key factors involved in the accumulation of the large LDL particles [2]. Traditional cardiovascular risk factors are implicated in the progression of endothelial dysfunction [2]. Following endothelial activation, an array of molecules (selectins, adhesion molecules) facilitate leukocyte rolling, adherence, and penetration in the subintimal space [3]. Those leukocytes are then differentiated into macrophages and engulf oxidized LDL, transforming into foam cells due to the presence of esterified cholesterol in lipid droplets [4]. Consequently, noxious inflammatory and oxidative responses arise [5,6], together with the activation and proliferation of vascular smooth muscle cells (VSMCs) in the media layer [7]. VSMCs in particular may also display phagocytic actions through the uptake of oxidized LDL, given that they are major contributors in atherosclerotic plaques [8,9].

**Figure 1:**
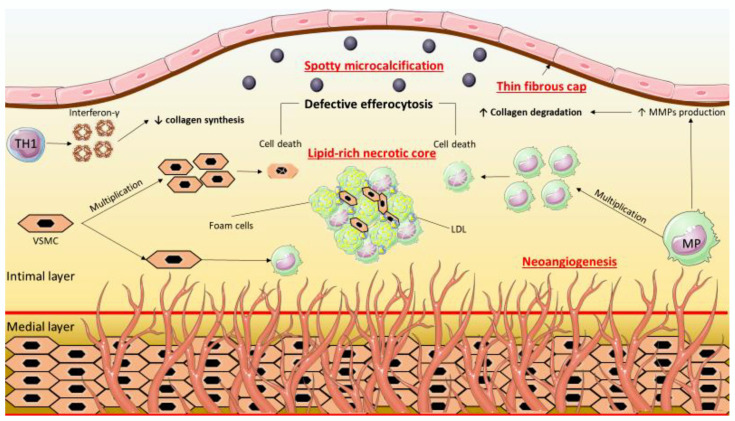
The characteristics of vulnerable plaque. A lipid-rich necrotic core, which is the outcome of macrophage multiplication and engulfment of LDL together with VSMC multiplication and differentiation into macrophages, may be detected. Moreover, decreased collagen synthesis and enhanced collagen degradation from the action of interferon-γ and MMPs, respectively, lead to the thinning of the fibrous cap. Other deleterious processes may also occur, such as the defective efferocytosis of cells, the spotty microcalcification as a result of inflammation and reduced collagen synthesis, and the vasa vasorum-derived neoangiogenesis. LDL: low-density lipoprotein, MMP: matrix metalloproteinase, MP: macrophage, TH1: type 1 T helper cell, VSMC: vascular smooth muscle cell.

Following their establishment, coronary atherosclerotic plaques progress through the continuous accumulation of lipids, the proliferation of VSMCs, and the decreased synthesis and increased degradation of collagen (Figure 1). The thinning of the fibrous cap follows, together with the apoptosis and defective efferocytosis, which contributes to the formation of a rich lipid core. Such plaques may also be termed as a thin-cap fibroatheroma (TCFA) [10]. Moreover, areas of calcification may also develop due to increased calcium deposition and decreased clearance. Spotty or microcalcification may lead to plaque instability [11]. Additionally, positive remodeling and plaque growth will promote neoangiogenesis, with the newly formed microvessels that stem from the vasa vasorum being another point of concern due to their ability to cause intraplaque hemorrhage [12].

## 3. Clinical Significance of Vulnerable Coronary Atherosclerotic Plaque

The correlation of high-risk features of VCAP with clinical events has been thoroughly described. In a landmark prospective study of 697 ACS survivors that underwent percutaneous coronary intervention (PCI) with intravascular ultrasound (IVUS), TCFAs were predictive of major adverse cardiovascular events (MACE) related to non-culprit lesions (HR 3.35, 95% CI 1.77–6.36, *p* < 0.001), together with plaque burden exceeding 70% and minimal luminal area ≤4 mm^2^ [13]. The prevalence TCFAs was significantly higher in patients with ruptured culprit coronary plaques compared to eroded ones, while the non-culprit lesions in patients with ruptured culprits were also characterized by TCFAs [14]. For ST-elevation myocardial infarction (STEMI) in particular, the non-culprit obstructive lesions consist of a TCFA in half of the cases, together with other vulnerability features [15]. Culprit ruptured plaques in individuals with STEMI possess more vulnerability features compared to culprit eroded plaques, possibly explaining the variability of clinical outcomes of these two distinct morphologies [16]. When comparing patients with STEMI and non-STEMI (NSTEMI), vulnerability features (microvessels, calcification, TCFAs) were more common in culprit and non-culprit lesions of STEMI patients [17]. Culprit TCFAs were independent predictors of STEMI and non-culprit TCFAs were associated with the incidence of MACE at the two-year follow-up [17]. In the group of patients with diabetes mellitus with optical coherence tomography (OCT)-derived fractional flow reserve-negative lesions, TCFAs were identified in 25% of the study population and were the strongest predictor of incident MACE consisting of cardiac mortality, target vessel myocardial infarction, clinically driven target lesion revascularization or unstable angina requiring hospitalization at 18 months (HR 5.12, 95%CI 2.1–12.3, *p* < 0.001) [18].

Recognition of the vulnerable plaque morphology has prompted research towards the interventional management of such lesions. It should be initially mentioned that complete revascularization was superior to culprit-only revascularization regarding the reduction of future adverse cardiovascular events in patients with STEMI in the Complete versus Culprit-Only Revascularization Strategies to Treat Multivessel Disease after Early PCI for STEMI (COMPLETE) trial [19]. A randomized control trial of patients with vulnerable non-obstructive lesions treated with either a bioresorbable vascular scaffold plus optimal medical therapy or optimal medical therapy alone found superior efficacy and similar safety of the intervention at a median follow-up of 4.1 years [20]. Ongoing studies should further clarify the importance of vulnerable plaque-guided percutaneous coronary intervention.

## 4. Non-Invasive Assessment of the Vulnerable Coronary Atherosclerotic Plaque

**Figure 2:**
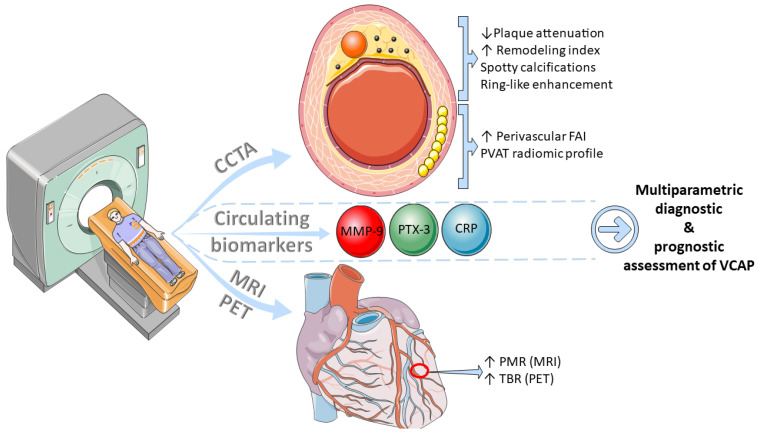
Multimodality non-invasive assessment of vulnerable coronary atherosclerotic plaques (VCAP). Combination of imaging (CCTA, MRI, or PET) and circulating markers of inflammation and extracellular matrix degradation could potentially enhance VCAP diagnostic and prognostic assessment. CCTA: coronary computed tomography angiography, CRP: C-reactive protein, FAI: fat attenuation index, MMP: matrix metalloproteinase, MRI: magnetic resonance imaging, PET: positron emission tomography, PMR: plaque-to-myocyte ratio, PTX: pentraxin, PVAT: perivascular adipose tissue, TBR: target-to-background ratio.

Although OCT and IVUS remain the gold-standard in the assessment of VCAPs, their invasive nature mandates the development of non-invasive modalities, including circulating biomarkers and imaging methods, which can promptly and accurately evaluate the presence of high-risk features and potentially aid the risk stratification and management of high-risk patients (Figure 2).

### 4.1. Circulating Biomarkers

Inflammation represents a cardinal feature of atherosclerosis [4] and, unavoidably, several inflammatory biomarkers have been assessed regarding plaque vulnerability (Table 1 and Table 2). Starting with the most studied inflammatory marker, C-reactive protein (CRP), its high levels were associated with the presence and the burden of TCFAs in patients with an ACS [21,22,23], as well as with plaque rupture [24]. The presence of increased CRP and high-risk features in OCT of patients hospitalized for an ACS may be an important prognostic clue for subsequent events [25]. However, other studies have found no association of CRP with lipid-rich plaques or with TCFAs [26,27], with the study of Koga et al. additionally pointing towards the association of pentraxin-3, another marker indicative of inflammation, with TCFAs instead of CRP [27]. A recent study further confirmed this hypothesis, with post-PCI pentraxin-3 being inversely correlated with fibrous cap thickness and positively correlated with lipid core length [28]. Critically, post-PCI pentraxin-3 values ≥ 4.08 ng/mL were identified as independent predictors of incident MACE [28]. Patients with STEMI and elevated pentraxin-3, together with plaque rupture or erosion, were at increased risk for future MACE [29].

Extracellular matrix (ECM) degradation is crucial in the formation of vulnerable plaques [30]. As a result, ECM biomarkers have been tested in this domain (Table 1 and Table 2). Among them, matrix metalloproteinase (MMP)-9 has been associated with the presence of TCFAs in the culprit lesion of patients with an ACS, with an area under the receiver operating characteristic curve (AUROC) of 0.83 and an optimal cutoff of 9.9 ng/mL [31]. MMP-9 levels ≥ 65.5 ng/mL were associated with ruptured plaques in patients with ACS [32]. In stable CAD patients with elevated lipoprotein (a), MMP-9 was independently associated with VCAPs [33]. Dynamic changes in MMP-9 have also been studied. A significant elevation in plasma MMP-9 is noted soon after plaque disruption in patients undergoing PCI, and is higher for those with an ACS or with lipid-enriched plaques [34].

While it appears that the role of biomarkers in the assessment of VCAPs is limited to date (Table 1 and Table 2), their combination into models might be of use. As shown in the study of Kook et al., such a model consisting of soluble lectin-like oxidized low-density lipoprotein receptor-1, MMP-9, white blood cell count, and the peak creatine kinase-myocardial band had decent AUROC (0.84), sensitivity (62.2%), and specificity (97.6%) for identification of plaque rupture in 85 patients with ACS, at a cutoff of 0.614 [35]. Furthermore, the addition of inflammatory biomarkers on top of imaging features of high-risk plaques may enhance the risk stratification for incident MACE [36]. Adequately sized clinical trials should be designed to assess the importance of combining circulating biomarkers with imaging features for the detection of VCAPs. Moreover, microRNAs are being investigated in the management of atherosclerotic diseases [37,38], and preliminary results have associated their levels with vulnerable plaque characteristics [39].

### 4.2. Computed Tomography Coronary Angiography

**Figure 3:**
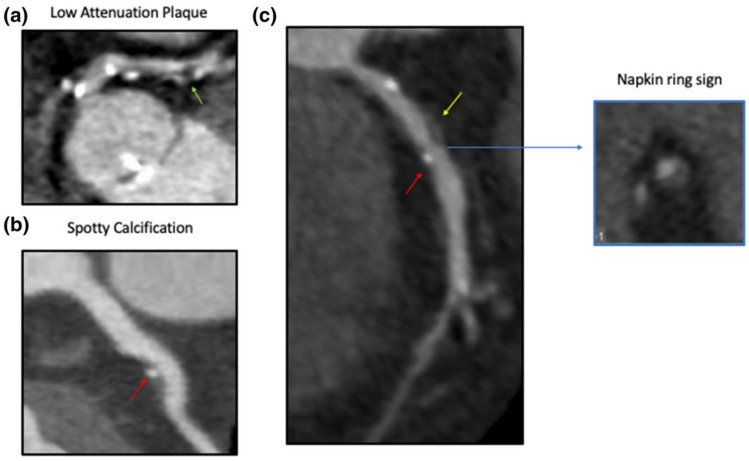
Coronary CT angiography findings of high-risk coronary atherosclerotic plaques. Certain identifiable patterns are indicative of a vulnerable plaque phenotype, namely (**a**) low plaque attenuation, (**b**) spotty calcification, and (**c**) the napkin ring sign. Reproduced with permission from Marwa Daghem et al. [41], British Journal of Pharmacology; published by John Wiley & Sons Ltd., 2021, used under Creative Commons CC BY 4.0 license. HU: Hounsfield units. Yellow arrow indicates positive remodeling, red arrow indicates spotty calcification, blue arrow indicates the napkin ring sign.

The use of multi-slice computed tomography (MSCT) for the identification of vulnerable plaque characteristics has been investigated thoroughly (Table 1 and Table 2, Figure 3). Initially, in the study of Pundziute et al., on 50 patients with stable CAD or ACS, the presence of a non-calcified or mixed plaque was a predominant finding in ACS compared to calcified plaques in stable CAD [40]. Interestingly, plaques characterized as TCFAs in IVUS were usually of mixed morphology in MSCT [40]. Similar results have been reported previously, with the presence of high-risk features (positive remodeling, spotty calcification, non-calcified plaques) being associated with ACS compared to stable CAD [41]. Small spotty plaque calcifications identified through coronary CT angiography (CCTA) were also correlated to the percentage of necrotic core and the prevalence of TCFA as assessed with IVUS [42].

Although a more precise characterization of plaque composition can be achieved with IVUS, a good correlation between the CT plaque classification and the IVUS-derived plaque composition has been noted [43]. When MSCT was compared to OCT in patients with ACS or stable CAD, important observations were reported. OCT-detected TCFAs in culprit lesions had a greater degree of positive remodeling and a lower attenuation value compared to non-TCFA culprit lesions [44]. Moreover, a ring-like enhancement in CT (plaque core with low CT attenuation surrounded by a rim-like area of higher CT attenuation) was common in TCFA, but with limited diagnostic accuracy (sensitivity: 44%, specificity 96%), however [44]. In the study of Ito et al., the assessment of coronary atherosclerotic plaques in 81 patients with clinically suspected CAD through OCT and MSCT demonstrated that an attenuation value of ≤62.4 Hounsfield Units (HU), a remodeling index (ratio of the outer cross-sectional vessel area at the site of the plaque divided by the outer area at the proximal reference site) ≥1.08, and a signet ring-like enhancement were independent predictors of OCT-defined TCFA in the multivariate analysis [45]. The diagnostic accuracy of plaque attenuation was the highest, with an AUROC of 0.859 [45]. The previously mentioned elements, together with the napkin-ring sign, were predictive of incident ACS in the study of Otsuka et al. [46]. In the study of Tomizawa et al., the investigators suggested that the low-attenuation plaque volume and remodeling index should be used as continuous values in conjunction with the napkin-ring sign in order to increase overall sensitivity and specificity to 94% and 91%, respectively [47]. Contrast/plaque attenuation ratios, created from CCTA for the characterization of each plaque component, were significantly correlated with IVUS-determined plaque component volumes [48]. A high necrotic core/fibrous plaque ratio may be related to IVUS-derived TCFA [49]. Increased epicardial fat volume and density have also been recognized as independent predictors of TCFAs [50,51].

Dual-source CT (DSCT) represents another non-invasive method of atherosclerotic plaque evaluation through the simultaneous image capture of two X-ray systems. As a result, enhanced temporal resolution and speed of acquisition can be achieved when paired with a significantly reduced radiation dose. Concerning VCAPs, they are associated with low CT values, with large cross-sectional plaque and lipid core areas. The differentiating ability of DSCT remains inadequate, however, with sensitivity and specificity of 73.1% and 94% in detecting TCFA, respectively [52]. A low-attenuation plaque volume greater than 8 mm^2^, derived from DSCT, had a remarkable diagnostic potential regarding IVUS-defined TCFA, with accuracy, sensitivity, and specificity of 91%, 84.6%, and 96.8%, respectively [53].

**Figure 4:**
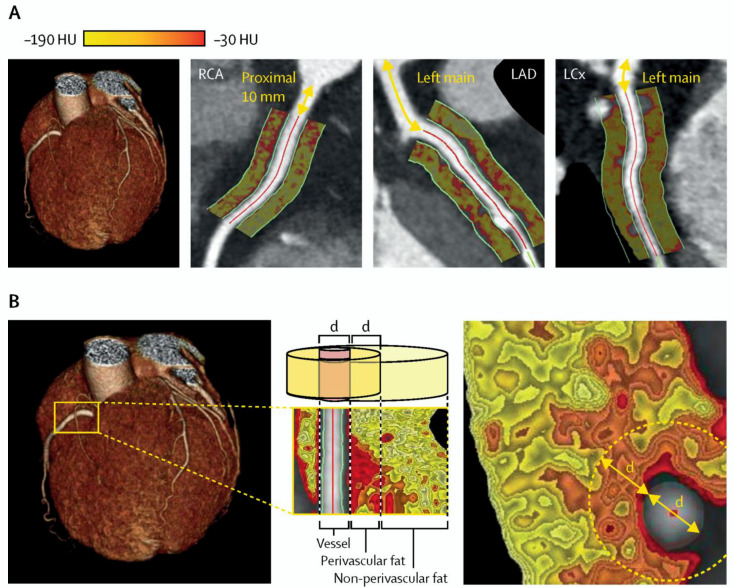
(**A**) Perivascular fat attenuation index (FAI) in the proximal area of the coronary vessels. (**B**) Perivascular FAI in the proximal segment of the right coronary artery (RCA). Perivascular fat occupies an area equal to the diameter of the vessel. Reproduced with permission from Oikonomou et al. [55], Lancet; published by Elsevier, 2018, used under Creative Commons CC BY 4.0 license. HU: Hounsfield units, LAD: left anterior descending artery, LCx: left circumflex artery.

The imaging of coronary perivascular adipose tissue (PVAT) with the so-called perivascular fat attenuation index (FAI) through CCTA deserves an honorable mention (Table 1 and Table 2, Figure 4). Perivascular FAI assesses adipocyte lipid content and size, indicative of vascular inflammation, with close correlation to inflammation detected by PET [54]. As a result, coronary inflammation and subclinical CAD may be identified. Perivascular FAI was associated with non-calcified atherosclerotic plaques and was increased in culprit lesions of patients with ACS [54]. These observations led to the hypothesis that the early detection of such lesions may be essential in identifying vulnerable plaques in vulnerable patients. This hypothesis was tested in the Cardiovascular RISk Prediction using Computed Tomography (CRISP-CT) study, involving 1872 and 2040 participants in the derivation and validation cohorts, respectively [55]. The perivascular FAI around the right coronary artery, with a cutoff of ≥−70.1 HU, was found to be predictive of all-cause (adjusted HR: 2.55, 95% CI 1.65–3.92, *p* < 0.001) and cardiac mortality (adjusted HR: 9.04, 95%CI 3.35–24.40, *p* < 0.001) and was, therefore, selected as the marker of coronary inflammation [55]. The results were confirmed in the validation cohort. Importantly, the addition of high perivascular FAI to a risk prediction model consisting of age, sex, cardiovascular risk factors (hypertension, hypercholesterolaemia, diabetes, smoking, and adipose tissue volume), the extent of coronary artery disease (modified Duke coronary artery disease index), and the number of high-risk plaque features added significant incremental prognostic value for all-cause (Δ_AUC_: 0.042, *p* = 0.0083) and cardiac mortality (Δ_AUC_: 0.075, *p* = 0.0069) [55]. The new predictive model incorporating perivascular FAI at the optimal cutoff was also more efficient in the classification of patients, as it was highly specific with excellent negative predictive value [55]. It should be noted that perivascular FAI was associated with clinical endpoints both for primary and secondary prevention across all of the examined subgroups [55]. In a post-hoc analysis assessing traditional high-risk plaque features (at least one of positive remodeling, low-attenuation plaque, spotty calcification, or napkin-ring sign) with FAI at the previously proposed cutoff, the presence of both high-risk plaque features and high FAI was associated with a 7.3-fold higher risk of cardiac death after adjustment for several factors compared, even when compared to the presence of high-risk plaque features alone [56]. The observations were similar, albeit attenuated, when FAI was assessed at the left anterior descending artery with a cutoff of ≥−79.1 HU [56]. Statin treatment has been found to decrease the perivascular FAI in high-risk lesions, representing an appealing approach for the monitoring of patient response and the assessment of the residual risk [57].

Perivascular FAI may also help discriminate the atherosclerotic changes with other inflammatory diseases such as myocarditis, which may present similarly to an ACS. However, perivascular FAI values are lower in the case of myocarditis compared to atherosclerosis, as recently demonstrated by Baritussio et al. [58]. Moreover, its use in chronic autoimmune inflammatory diseases needs to be elucidated further, since patients with psoriasis had significantly lower vascular inflammation assessed by perivascular FAI [59], opposed to the common belief that chronic low-grade inflammation in such pathologic states leads to a greater extent of inflammatory atherosclerotic changes. Although no differences were noted regarding the use of biologic therapy or statins in this study [59], Elnabawi et al. had previously shown a decrease in perivascular FAI following the use of biologic therapy in patients with moderate-to-severe psoriasis [60].

CCTA radiomics may be an important next step in advancing the recognition of vulnerable plaques via CT (Table 1 and Table 2), being superior in diagnostic accuracy compared with the conventional high-risk plaque features from IVUS, OCT, or positron emission tomography (PET) [61,62,63]. Radiomic profiling of PVAT remodeling alterations has also been investigated. Adipose tissue wavelet-transformed mean attenuation was sensitive in detecting PVAT inflammation, while features of radiomic texture were related to PVAT fibrosis and vascularity [64]. As far as their relationship with MACE is concerned, the development of a machine learning algorithm consisting of the fat radiomic profile was derived from a training cohort and then validated in 1575 individuals of the Scottish Computed Tomography of the Heart (SCOT-HEART) trial, improving prediction of incident MACE compared to standard features assessed by CCTA (Δ_C-statistic_: 0.126, *p* < 0.001) [64]. Moreover, the fat radiomic profile was increased in ACS patients compared to matched controls and it remained unchanged after a six-month follow-up, possibly indicating permanent changes in PVAT [64]. Ultimately, the recently developed CaRi-Heart^®^ device, incorporating the evidence from the previously mentioned studies, drastically improved the risk stratification of patients compared to conventional models (Δ_C-statistic_: 0.149, *p* < 0.001 in the validation cohort) [65].

### 4.3. Magnetic Resonance Imaging

Despite the fact that cardiac magnetic resonance imaging (cMRI) is not widely adopted in the evaluation of VCAP, considerable scientific research has been performed in this domain. Early studies have shown the MRI-assessed area of plaque tissue components (lipid-rich necrotic core, calcium) correlated with the histopathologic evaluation. Importantly, those two components could be reliably differentiated from fibrous tissue. The histopathologically defined vulnerable plaque was associated with a large lipid area and reduced minimal fibrous cap thickness in MRI [66]. The local stress/strain pattern in areas of TCFAs was proposed as another index of MRI-defined plaque vulnerability [67]. Plaque wall stress was assessed by Huang et al. using ex-vivo MRI in coronary plaques of 12 deceased patients with the use of three-dimensional fluid-structure interaction models, thus calculating the critical plaque wall stress [68]. This parameter was significantly increased in patients that died from CAD-related causes compared to the control group, while the plaque burden did not differ significantly [68]. Segmental pericoronary epicardial adipose tissue volume, quantified by cMRI, has been associated with CT-derived vulnerability features such as low attenuation and non-calcified or mixed morphology [69].

Moving to non-contrast T1-weighted images, in a prospective study of 568 patients with suspected or known CAD, the presence of high-intensity plaques [plaque-to-myocardium signal intensity ratio (PMR) ≥ 1.4] together with a history of CAD was independently associated with incident coronary events (HR: 3.96; 95% CI: 1.92–8.17, *p* < 0.001) [70]. Utilizing this approach in 77 patients with stable CAD undergoing PCI, Hoshi et al. correlated high-intensity plaques based on the above-mentioned cutoff to IVUS-derived characteristics of vulnerable plaques [71]. The presence of a high-intensity plaques was also associated with periprocedural myocardial injury [71]. In another study, high-intensity signal plaques with PMR > 1 were characterized based on their location as intrawall or intraluminal, which had important morphological implications [72]. Specifically, intrawall high-intensity signal plaques had macrophage accumulation in the absence of calcifications, whereas intraluminal plaques were more commonly met with thrombi and intimal microvessels [72]. Compared to OCT, PMR as a continuous variable was linearly correlated with the number of high-risk plaque features of the culprit lesion [73]. Among those high-risk features, non-calcified plaque, thrombus, and intimal vasculature were independently associated with PMR [73]. Intensive 12-month statin therapy led to the reduction of PMR in high-intensity plaques [74], thus providing an additional role in the monitoring of patients.

As far as contrast-enhanced cMRI is concerned, early contrast enhancement of a coronary plaque may also be a sign of vulnerability, as it is more frequently encountered in cases of unstable angina pectoris compared to patients with stable CAD [75]. Moreover, delayed contrast enhancement with the use of contrast-to-noise ratio (CNR) was significantly higher in culprit lesions compared to non-culprit lesions [76]. Gadofosveset-enhanced cMRI (GE-cMRI) could identify and exclude culprit lesions in ACS or CAD patients (sensitivity: 82%, specificity: 83%), while the areas where TCFAs were detected through OCT were characterized by increased CNR [77]. When comparing GE-cMRI with T1-weighted cMRI in patients with clinical suspicion of CAD, hemodynamically significant lesions with a quantitative flow reserve < 0.8 had higher CNR lesion only in GE-cMRI [78].

### 4.4. Nuclear Imaging

The increasing frequency in the use of nuclear imaging techniques in cardiovascular diseases has not spared the assessment of VCAP. Initial studies were conducted in cancer patients, with 18-fluorodeoxyglucose (FDG) PET/CT detecting significant correlations of target-to-background ratio (TBR) in the region of the left anterior descending artery with cardiovascular risk factors, pericardial fat volume, and calcified plaque burden [79]. However, myocardial uptake of FDG limited its applicability in the entire patient population (Table 1 and Table 2) [79]. Myocardial FDG uptake could be diminished through consumption of a low carbohydrate, high-fat meal the night before the procedure [80], a finding which was confirmed in a randomized trial [81]. However, patients with diabetes mellitus should be handled with caution, since regular dietary recommendations are usually ineffective, as these patients may not be able to produce adequate insulin in response to glucose loading. Therefore, techniques such as the euglycemic-hyperinsulinemic clamp should be applied for adequate image quality and results [82].

18-sodium fluoride (NaF) is an alternative tracer that has been used, and is indicative of calcification and macrophage activity. As there is limited myocardial uptake through the use of this tracer, motion correction techniques have been additionally applied to enhance coronary artery plaque visualization, with an encouraging 46% reduction of image noise being achieved [83]. Triple-gated corrections may further augment the reproducibility of the examination [84]. As far as atherosclerotic plaque assessment is concerned, NaF uptake was increased in patients with CAD and correlated with calcium score, while the 18-FDG uptake did not differ according to CAD status [85]. A few vulnerable plaques were detected in diabetics without known CAD, with a TBR cutoff ≥ 1.5 [86]. The prevalence of fluoride-positive plaques was higher in patients after an ACS compared to stable CAD in another study [87]. Other than TBR, the efficiency of coronary microcalcification activity (CMA) across the entire coronary circulation has been tested in patients with recent ACS and multivessel CAD [88]. Both CMA and TBR were increased in low-attenuation plaques compared to the rest, but a CMA threshold >0 was superior in detecting the low-attenuation plaques compared to a TBR > 1.25, with remarkable sensitivity and specificity (93.1% and 95.7%, respectively) [88].

Using tracers that target specific plaque components is also being investigated. Interest has been shown towards (68)Ga-DOTATATE, a tracer that binds to somatostatin receptor 2 that is expressed in macrophages. In patients with neuroendocrine tumors, the use of this tracer in PET/CT demonstrated significantly higher TBR in atherosclerotic plaques compared to normal coronary arteries [89]. Through the use of a tracer that targets vascular cell adhesion molecule-1, in-vivo PET/CT imaging of the aorta in murine models was successful in diagnosing atherosclerotic lesions and their extent [90]. Furthermore, utilization of a selective radiotracer for MMP-13 led to superior identification of plaques with MMP-13 expression, indicative of extracellular matrix remodeling and, thus, potentially vulnerable [91]. Additionally, (68)Ga-pentixafor is known for its binding ability to the CXC-motif chemokine receptor 4 (CXCR4), which is implicated in atherosclerosis. Following PET/CT imaging with this radiotracer, an increased uptake was noted in calcified plaques and in patients with an increasing number of cardiovascular risk factors [92]. Lastly, the use of a novel glycoprotein IIb/IIIa-receptor radiotracer, 18F-GP1, has been recently evaluated in 44 patients after myocardial infarction. Culprit vessels had higher uptake of the radiotracer compared to non-culprits, whose uptake was similar to that of controls. The optimal cutoff of the maximum TBR for the culprit vessel was reported at 1.20 (Sensitivity: 60%, Specificity: 97%) [93].

Even though PET/CT has received most of the attention, hybrid PET/MRI imaging methods may be considered as an option, even though their use has been mostly experimental to this point, aiming at improving image quality [94]. In the only available clinical study (Table 1 and Table 2), the use of 18-NaF in gadobutrol-enhanced PET/MRI led to the identification of TCFAs and lipid cores in segments with TBR > 1.28 and >1.25, respectively [95]. Interestingly, CNR was correlated with calcified TCFA in cases of TBR > 1.28 [95]. Awareness in the field of nuclear imaging of atherosclerosis is rapidly growing, with the upcoming clinical studies being eagerly awaited. Finally, it should be stressed that nuclear imaging studies have no relevant contraindications in subjects with chronic kidney disease, in contrast to CCTA and cMRI, where there is a concern of serious renal complications such as CI-AKI and nephrogenic systemic fibrosis (Table 1).

## 5. Non-Invasive Assessment of VCAP: Current State

To conclude, the presented evidence concerning the progress of non-invasive modalities in the assessment of VCAPs indicates the extensive knowledge we have attained with regard to the process of coronary atherosclerosis. Identification of such adverse plaque characteristics may thus represent an appealing option in the holistic management of patients with CAD by providing incremental prognostic information and tailoring the therapeutic approach. Published studies have shown a good correlation with invasive methods or even plaque histology. On top of that, abnormalities in circulating inflammatory and extracellular matrix degradation biomarkers could indicate an additional risk marker. However, the lack of large-scale, multicenter randomized clinical trials and registries is a deterring factor for the widespread implementation of these modalities in everyday clinical practice. Moreover, uncertainties remain regarding the optimal imaging method of choice, with most data stemming from CCTA studies. Limited evidence is available from nuclear imaging studies, which may also face certain limitations concerning myocardial uptake and motion correction that ought to be resolved. Therefore, upcoming studies should be adequately designed to provide the needed answers in the existing evidence gaps and prove the incremental value of the non-invasive, multimodality assessment of VCAPs.

## Figures and Tables

**Table 1:** Comparison of non-invasive modalities for vulnerable plaque assessment.

	Biomarkers	CCTA	cMRI	PET
Vulnerable plaque characteristics	↑ hsCRP ↑ PTX-3 ↑ MMP-9	↓ Plaque attenuation ↑ Remodeling index ↑ perivascular FAI Ring-like enhancement Spotty calcifications	↑ PMR ↑ CNR	↑ TBR ↑ CMA
**Clinical correlates**				
Correlation with OCT/IVUS-derived plaque characteristics	+	+++/++++	+++	++
Gaps in evidence	Ideal sampling site (Peripheral vein, lesion location)	Radiomic-based approaches	Definite cutoffs	Ideal radiotracer PET/CT or PET/MRI Lack of definite cutoffs Limitation of myocardial uptake in FDG-PET Motion correction
**Modality features**				
Accessibility	++++	+++	++	+
Cost	+	++	+++	++++
Side-effects	-	Radiation exposure Anaphylactic reaction to IVCM CI-AKI	Anaphylactic reaction to IVCM Nephrogenic systemic fibrosis	Anaphylactic reaction

CCTA: coronary CT angiography, CI-AKI: contrast-induced acute kidney injury, CMA: coronary microcalcification activity, cMRI: cardiac magnetic resonance imaging, CNR: contrast-to-noise ratio, FAI: fat attenuation index, FDG: fluorodeoxyglucose, hsCRP: high sensitivity CRP, IVCM: intravenous contrast medium, IVUS: intravascular ultrasound, MMP-9: matrix metalloproteinase-9, OCT: optical coherence tomography, PET: positron emission tomography, PMR: plaque-to-myocardium ratio, PTX-3: pentraxin-3, TBR: target-to-background ratio. ↑ indicates increased, ↓ indicates decreased, number of + indicates the strength of the correlation.

**Table 2:** Available non-invasive modalities in the assessment of vulnerable coronary atherosclerotic plaques.

**Biomarkers**		
CRP	**↑ levels associated with**	→ ↑ prevalence and burden of TCFAs → Plaque rupture → ↑ Incidence of subsequent events
Pentraxin-3	↑ levels associated with	→ ↑ TCFAs → ↑ lipid core length → ↓ Fibrous cap thickness → Ruptured vs. eroded plaques → ↑ incidence of MACE
MMP-9	↑ levels associated with	→ TCFA in culprit lesions → Ruptured plaques/plaque disruption
**CCTA features**		
Spotty calcifications	Associated with	→ Percentage of necrotic core → Prevalence of TCFA
↓ Plaque attenuation ± Positive remodeling ± Ring-like enhancement	Associated with	→ Prevalence of TCFA in culprit lesions → Plaque component volumes → ↑ incidence of ACS
Necrotic core/fibrous plaque ratio	Associated with	→ Prevalence of TCFA
Perivascular FAI	↑ values (≥−70.1 HU) associated with	→ Culprit lesions → Predictive of all-cause and cardiac mortality → Incremental prognostic value on top of clinical and other imaging features → 7.3-fold higher risk of cardiac death in the presence of ↑ FAI and traditional high-risk plaque features
PVAT radiomic profile	Associated with	↑ prediction of incident MACE
**cMRI features**		
PMR	↑ values (≥1.4) associated with	→ Vulnerable plaque characteristics and their number → Periprocedural myocardial injury
CNR	↑ values associated with	→ Culprit lesions → Prevalence of TCFA
**Nuclear imaging features**		
TBR	↑ values (>1.25) associated with	→ Low-attenuation plaques → Prevalence of TCFA and lipid core
CMA	↑ values (>0) associated with	→ Low-attenuation plaques

ACS: acute coronary syndrome, CCTA: coronary computed tomography angiography, CMA: coronary microcalcification activity, cMRI: cardiac magnetic resonance imaging, CNR: contrast-to-noise ratio, CRP: C-reactive protein, FAI: fat attenuation index, HU: Hounsfield units, MACE: major adverse cardiovascular events, MMP: matrix metalloproteinase, PMR: plaque-to-myocardium ratio, PVAT: perivascular adipose tissue, TBR: target-to-background ratio, TCFA: thin cap fibroatheroma. ↑ indicates increased, ↓ indicates decreased.

## Data Availability

Not applicable.
